# The iPeer2Peer Program: a pilot randomized controlled trial in adolescents with Juvenile Idiopathic Arthritis

**DOI:** 10.1186/s12969-016-0108-2

**Published:** 2016-09-02

**Authors:** Jennifer Stinson, Sara Ahola Kohut, Paula Forgeron, Khush Amaria, Mary Bell, Miriam Kaufman, Nadia Luca, Stephanie Luca, Lauren Harris, Charles Victor, Lynn Spiegel

**Affiliations:** 1 Lawrence S. Bloomberg Faculty of Nursing, University of Toronto, Toronto, Canada; 2 The Hospital for Sick Children, Child Health Evaluative Sciences, Toronto, ON Canada; 3 Department of Psychology, The Hospital for Sick Children, Toronto, ON Canada; 4 University of Ottawa, Faculty of Health Sciences, School of Nursing, Ottawa, ON Canada; 5 Department of Rheumatology, Sunnybrook Health Sciences Centre, Toronto, ON Canada; 6 Department of Adolescent Medicine, The Hospital for Sick Children, Toronto, ON Canada; 7 Department of Medicine, Rheumatology, Alberta Children’s Hospital, Calgary, AB Canada; 8 University of Toronto, Institute for Clinical Evaluative Sciences, Toronto, ON Canada; 9 Department of Rheumatology, The Hospital for Sick Children, Toronto, ON Canada

**Keywords:** Juvenile Idiopathic Arthritis, Social support, Adolescents, Randomized controlled trial

## Abstract

**Background:**

Adolescents with Juvenile Idiopathic Arthritis (JIA) are at risk for physical, emotional, social and role challenges that negatively impact quality of life. Peer mentoring has been shown to improve positive health behaviours in adolescents with chronic disease while simultaneously providing social support. The objectives of this paper are to examine the feasibility and acceptability of an online peer mentoring program (iPeer2Peer Program) for adolescents with JIA.

**Methods:**

The iPeer2Peer program was examined using a waitlist pilot randomized control trial (RCT). Participants were randomly allocated to the intervention or wait-list control group via a secure, web-based randomization service. Health care providers and investigators were blinded to participant group allocation. Trained peer mentors (16–25 years; successfully managing their JIA) were matched to participants (12–18 years; diagnosed with JIA) randomized to the intervention group to provide peer support and education for effective self-management of JIA. Participant-mentor pairings connected ten times over 8 weeks using Skype video calls. Primary outcomes focused on implementation (i.e. measures of feasibility and acceptability). Secondary outcomes focused on effectiveness (i.e. measures of self-management, self-efficacy, pain, social support and quality of life).

**Results:**

Thirty adolescents (mean age 14.3 ± 1.7 years, 97 % female) completed the RCT (intervention *n* = 16, control *n* = 14). *Primary outcomes:* One third (32 %) of adolescents approached agreed to participate, completed baseline measures and were randomized. Half of pairings completed ten calls within 8 weeks. Average call length was twice the required amount with call lengths of 44.72 ± 15.76 min. Participants reported satisfaction with the program and all reported that they would recommend it to their peers. Participants’ mean engagement level with the program was 8.53/10 (range = 7–10). *Secondary outcomes:* Participants who completed the iPeer2Peer Program demonstrated improvements in their perceived ability to manage JIA (*p* < 0.04), compared to controls. No adverse events were reported.

**Conclusion:**

The iPeer2Peer Program is a promising intervention that improves acceptability of self-management and peer support treatments for adolescents with JIA. By using the Internet to connect mentors to adolescents with JIA it may also improve accessibility to these resources. Findings will be used to adapt the program and refine the methodology for a full-scale RCT.

**Trial registration:**

ClinicalTrials.gov Identifier: NCT01986400. Registered November 11, 2013.

## Background

Juvenile Idiopathic Arthritis (JIA) is the most common pediatric rheumatic disease with a prevalence of 132 per 100,000 children [1]. JIA negatively impacts all aspects of health-related quality of life (HRQL) including physical, emotional, social and role challenges [2–7]. Adolescents living with JIA may be disadvantaged in comparison to healthy same age peers since common developmental goals, such as developing one’s sense of self and becoming independent, become more challenging to achieve as they manage symptoms, therapies, appointments and procedures [8–10]. When compared to healthy peers, adolescents with JIA report increased depression, anxiety, poor self-esteem and social disruption [2, 5–7]. A metasynthesis of qualitative studies of the experiences of adolescents living with JIA found six major domains in which JIA impacted their life: aversion to being different, striving for normality, stigma and misunderstanding, suspension in uncertainty, managing treatment and a desire for knowledge [11]. The findings from the metasynthesis suggests that social differences (e.g., feeling different from peers, healthy peers not understanding their challenges) contribute to negative experiences for adolescents with JIA. Peer mentoring, therefore, is one method that is ideally suited to meet the unique emotional, social and developmental needs of adolescents with JIA.

Peer mentoring in health care is an explicit form of peer social support in which a trained peer mentor provides emotional, appraisal, and informational support to another person living with a similar condition [12]. Peer mentoring has been associated with improved diverse health outcomes [13–15]. However a systematic review on interventions to improve psychological adaptation to chronic disease by adolescents concluded that the efficiency of peer support groups requires more research; in particular how to achieve greater reach and adoption using novel information and communication technologies [16]. Research suggests that only 25 % of youth go online to visit forums or contact other youth with JIA and that these sites are not perceived as relevant (rated as a median of 1.0 out of 10 on a scale of relevance) [17]. There is a clear need to develop acceptable and accessible social supports for adolescents with JIA.

In order to meet the informational, physical, emotional and social needs of adolescents with JIA, an online peer mentoring intervention, the iPeer2Peer Program, was developed [13, 18]. The iPeer2Peer Program focuses on pairing an adolescent living with JIA with a trained young person successfully managing JIA. The peer mentors endeavor to act as positive role models to help reinforce self-management of JIA while also providing essential social support to adolescents with JIA. Moreover, acting in a peer mentor role may positively impact the peer mentors’ own self-efficacy and self-management skills [19]. The iPeer2Peer Program has been successfully tested in pediatric chronic pain populations [13]. The aims of this study were to determine the feasibility and acceptability of the iPeer2Peer Program in adolescents with JIA as well as to explore effectiveness outcome measures for the purposes of refining the program and methodology before a full scale randomized controlled trial (RCT).

## Methods

### Study design

A waitlist RCT design was used to examine the feasibility, acceptability, and effectiveness of the iPeer2Peer program in adolescents with JIA.

### Participants

Participants were recruited from a rheumatology clinic in one large urban Canadian pediatric tertiary hospital. Adolescents were eligible for participation if they were (a) 12–18 years old, (b) diagnosed with JIA according to the International League of Associations for Rheumatology (ILAR) criteria [20], (c) able to speak and read English such that they could carry a conversation with their mentors, (d) able to access the Internet on a computer compatible with Skype software, and (e) willing and able to complete online measures. Adolescents were excluded if they had (a) significant cognitive impairments or (b) major co-morbid illnesses (i.e., psychiatric conditions such as schizophrenia, bipolar disorder) likely to influence assessment of HRQL, or were (c) participating in another peer support or self-management intervention.

### Procedures

**Fig. 1:**
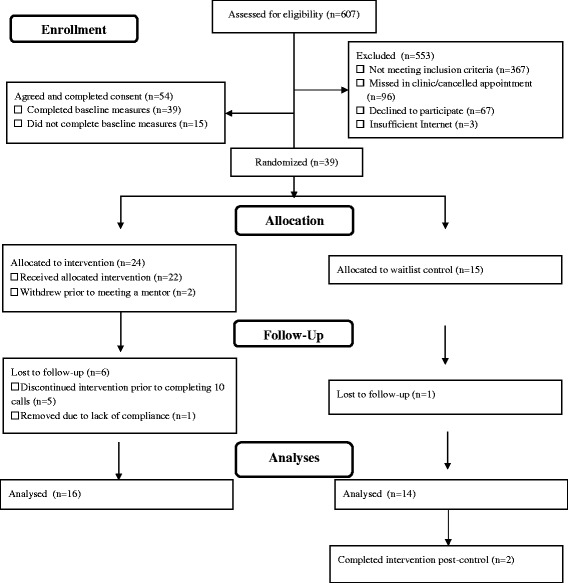
Study flow diagram

Following institution Research Ethics Board approval (#1000038163), eligible adolescents who had a scheduled appointment at the rheumatology clinic at the tertiary care hospital were approached to participate by a Clinical Research Project Coordinator (CRPC). If interested, the CRPC provided further information and obtained informed consent. The CRPC obtained baseline data from participants (demographic and disease-related characteristics) and emailed participants online pre-intervention measures. Once these measures were complete, the participants were randomly allocated to the intervention or wait-list control group. A secure, web-based randomization service (www.randomize.net) was used to allocate participants to the trial groups. The CRPC then contacted participants to inform them of their group assignment and instruct them on the procedures to be followed (See Fig. 1 for trial schema).

#### Experimental group

In addition to standard medical care, adolescents in the experimental group received the iPeer2Peer Program and were sex-matched with a trained peer mentor. The mentoring program consisted of 10 sessions of 20–30 min Skype video calls conducted over eight weeks. Participant-mentor pairings had two sessions per week for the first two weeks, and one session per week for six weeks. Mentoring sessions occurred only during the scheduled times and participant-mentor contact outside of the mentoring sessions was discouraged. If pairings missed a call, they were sent email reminders from the CRPC to reschedule. If a subsequent call was missed the CRPC would follow-up via telephone to remind the mentor and participant of the call schedule and/or offer problem solving support (e.g., advice to schedule a consistent weekly call time). The topics of each conversation were not predetermined or dictated by a protocol. Call content was open ended and tailored to each participant’s expressed needs and desires during the calls. However, mentors were trained (see below) in a standardized training protocol to focus the calls on providing social support and encouraging participants to develop and engage in self-management skills, and support the practice of these skills. Peer mentors were compensated $20 per call.

All calls were audio-recorded and uploaded to a secure research server. Mentors were trained to flag concerns that needed immediate follow-up by a member of the research team (e.g., self-harm). To ensure safety, a member of the research team reviewed all calls within 48 h. During this review, a log of all topics of conversation in each call was kept in order to determine the content of the iPeer2Peer Program for each participant. Although the goal of the iPeer2Peer program was to train mentors who would be able to provide self-management and social support individualized to the circumstances of each participant, the log was used in order to determine the proportion of calls that discussed various disease-related topics (e.g., self-management, coping, impact of JIA on relationships, school and work). This log was used as a measure of program adherence and mentor reliability during the mentoring calls.

##### Peer mentor selection and training

In addition to having a diagnosis of JIA, peer mentors were nominated by their health care team based on maturity, emotional stability, and verbal communication skills. Mentors were then screened by the research team based on the following criteria: 1) 16–25 years old, 2) diagnosed with JIA, 3) self-reported adherence to current treatment plan (minimum 80 % compliance), 4) self-reported successful transfer to an adult care facility, 5) do not currently meet DSM-IV-TR [21] criteria for a psychological or psychiatric illness as determined by health care team, 6) self-reported “high” self-efficacy in their ability to manage JIA, and 7) willingness and ability to complete peer mentor training.

##### Peer mentor training

Peer mentor training involved both self-directed in home reading and an on-site 2.5 day training session facilitated by a health psychologist and an adult living with JIA who’s occupation involves training young adult peer mentors. In-class training comprised of lectures, active group discussion, case examples, small group activities and role-play activities. All peer mentors received a manual, which included all training materials, additional resources (e.g., information sheets on pacing, relaxation, reputable online resources) and reading lists. The training manual provided suggested topics of conversation for mentorship calls (e.g., coping strategies, lifestyle management, communicating effectively with health care team), advice on structuring conversations and guides to redirect conversation to self-management topics. Peer mentors also had access to research staff throughout the study and, if needed, mentors were given additional training to help improve mentoring skills. Training was based on a conceptual framework and focused on providing peer mentors with the skills necessary to provide informational, emotional and appraisal support to adolescents [12]. The iPeer2Peer Program has also been tested in a group of adolescents with chronic pain. No adaptations or changes were made to the peer mentor training. For more detailed information on peer mentor selection and training, please refer to our study in the adolescent chronic pain sample [13].

#### Control group

The control group received standard care but without the iPeer2Peer Program. Participants in the control group were offered the iPeer2Peer Program after completion of post-control outcome measures. If they chose to enroll in the iPeer2Peer Program, they were asked to complete outcome measures a third time upon completion of the program.

### Outcome measures

Primary outcomes and endpoints of this study focused on implementation of the iPeer2Peer Program as measured by: (a) recruitment and withdrawal rates, (b) adherence with the iPeer2Peer Program (defined as 100 % when the participant completes ten calls over 8 weeks), (c) proportion of completed questionnaires (defined as 100 % when all measures completed), and (d) engagement and satisfaction with the iPeer2Peer Program as measured through a semi-structured phone interview with participants following involvement in the iPeer2Peer Program (including asking participants to rate level of engagement on a scale of 0–10 with higher scores reflecting higher engagement with the iPeer2Peer Program).

Secondary outcomes of this study focused on the effectiveness of the iPeer2Peer program. Given that the focus of this present study was feasibility, the secondary outcome measures were exploratory to examine variances to help determine appropriate outcomes and sample size for future full-scale RCTs. Measures were chosen based on the results of a systematic review of existing peer support interventions in youth with chronic disease [14]. Participants completed four measures at baseline and completion of the study to capture self-management (Medical Issues, Exercise, Pain and Social Support Questionnaire [MEPS] [22]), pain (Recalled Pain Inventory [RPI] [23]), perceived social support (social support subscale of the MEPS) [22], self-efficacy (Children’s Arthritis Self-Efficacy [CASES] [24]) and HRQL (PedsQL Arthritis Module) [25]. All measures have evidence of reliability and validity in samples of adolescents with JIA. Participants were offered $15 for completion of outcome measures at baseline and at program completion.

Adolescent participants (i.e., mentees) who completed the iPeer2Peer program were invited to complete a 15-min semi-structured telephone interview describing the strengths and weaknesses of the program. A trained research assistant who was not involved in the implementation of the iPeer2Peer program conducted all semi-structured interviews.

### Data analysis

Means and standard deviations were used to summarize continuous factors and frequencies and percentages were used to summarize categorical factors. The primary and secondary continuous outcomes were compared across experimental and control groups using marginal linear models assuming an exchangeable covariance matrix to account for the repeated measurements of some participants (i.e., control group participants completing the iPeer2Peer program following initial follow-up period). Outcome measures were modeled onto intervention status, and adjusted for period of observation (i.e., two waitlist control participants took part in the program following their primary period of observation and had a second period of observation while in the program) and baseline score of the same measure (e.g., PedsQL pain). The semi-structured individual interviews were audio taped, transcribed and analyzed using qualitative content analysis [26].

## Results

Thirty adolescent participants (96.7 % female) aged 14.3 ± 1.7 years (range = 12–17 years) completed the trial. There were no significant differences in the age of adolescents who chose to participate in the study versus those who declined participation (14.9 ± 1.8 years; range = 12–17 years). Two adolescents completed the intervention after completing participation in the control group (see Table 1). Outcome analysis controlled for repeated measurement in these two adolescents.

**Table 1:** Demographic and disease characteristics of participants and mentors

Characteristic	iPeer2Peer program	Control
	*n* (%)	*n* (%)
Participants (total *n* = 30)	18^a^	14
Age, mean ± SD years	14.11 ± 1.53	14.42 ± 2.04
Grade, mean ± SD	9.11 ± 1.57	9.50 ± 2.21
Sex		
Female	17 (94)	14 (100)
Male	1 (6)	0 (0)
Diagnosis		
Polyarthritis (RF positive)	3	3
Polyarthritis (RF negative)	3	3
Polyarthritis (RF status unknown)	1	0
Oligoarthritis	5	5
Psoriatic Arthritis	6	2
Juvenile Enthesitis-related Arthritis	0	1
Systemic Arthritis	0	0
Disease Activity, mean ± SD, range = 0–10^b^	1.71 ± 2.81	1.27 ± 1.10
Duration since diagnosis, mean ± SD years; Duration since diagnosis, range	7.86 ± 4.85	7.30 ± 4.78
	<1 month–14.16 years	1.25–15.65 years
Mentors (*n* = 6)		
Age, mean ± SD years	18.88 ± 1.48	
Sex		
Female	5 (83)	
Male	1 (17)	
Diagnosis		
Polyarthritis (RF negative)	3 (50)	
Oligoarthritis	1 (17)	
Psoriatic Arthritis	1 (17)	
Systemic Arthritis	1 (17)	
Duration of illness, mean ± SD years	8.52 ± 6.40	
Number of mentees, mean ± SD	3.60 ± 2.97	
Number of mentees, range	1–8	

*Notes.* ^a^All participants, including the post control iPeer2Peer Program participants (*n* = 2). ^b^Disease activity is measured via a physician global assessment visual analog scale

### Primary outcome analysis

The primary outcomes for this study focused on the feasibility and acceptability of the iPeer2Peer program. Parameters explored included recruitment, program adherence, topics of conversation and satisfaction with the iPeer2Peer Program.

#### Recruitment

Of those participants who were approached, eligible and able to participate, 44.63 % (*n* = 54) agreed and completed consent, while 32.23 % (*n* = 39) completed baseline measures and were randomized. Of those who agreed to participate, 5.1 % (*n* = 2) withdrew after completing baseline measures, but prior to beginning the intervention. Time commitment was the stated reason for withdrawing from the program. There was less than 1 % missing data in the study measures. Three adolescents had to decline participation despite interest in the iPeer2Peer Program due to lack of sufficient and reliable Internet access to run Skype at home or at a nearby location (e.g., library). See Fig. 1 for study flow, including data on recruitment and dropout.

#### Program adherence

Data from all participants in the intervention group who completed a minimum of one call were included in this analysis (*n* = 22). Fifty-five percent (*n* = 12) of participants completed all 10 calls, while the remaining completed, on average, 7.58 calls ± 3.05 (range = 1–10 calls). Of the 12 participants who completed 10 calls, 58 % of them were able to complete the program within the requested 2 months (60 days). Mean program length was 80.79 ± 53.98 days (range = 1–210 days). Of the participants who had a minimum of two calls (*n* = 21), pairings had video calls every 10.87 ± 5.52 days. The vast majority (93.4 %) of calls adhered to minimal call length of 20 min with average call lengths twice that amount (44.72 ± 15.76 min, range 10.38–84.33 min). Of those participants who did not complete 10 calls, 94.74 % of their calls were adherent to call length with a mean of 38.53 ± 13.78 min long (range = 17.67–72.48 min). The most common reason for rescheduling mentorship calls were tests, assignments, or non-JIA related sickness in either participant or mentor.

With respect to male participation, of those who were approached, eligible and able to participate, three males were randomized to the intervention. One male withdrew prior to being matched to a mentor. The remaining male participants completed seven and five calls and call length, on average, was 29.76 ± 5.62 min long (range = 23.28–39.32 mins).

**Fig. 2:**
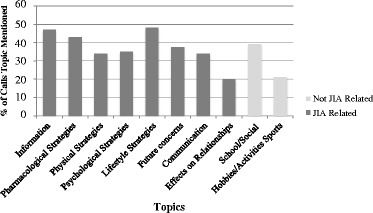
Topics discussed during ipeer2peer program video calls

Call log data demonstrated that participant-mentor pairings spoke in every call about issues related to JIA but also often spoke about issues related to their life in general. The most common topics of conversation included lifestyle management (e.g., sleep hygiene, time management, staying motivated and following clinician recommendations), information about JIA and issues around medications. Notably, 37.5 % of calls involved conversations related to concerns for the future, whether related to education, occupation or relationships. Approximately 12.5 % of calls involved a structured goal-setting and action-planning task (e.g., use of the SMART goals). See Fig. 2 for a summary of topics of conversation discussed during a minimum of 20 % of the calls.

#### Satisfaction

During post-iPeer2Peer program telephone interviews of adolescents who were matched to a mentor (*n* = 17, one participant did not complete interview due to scheduling difficulties), participants reported mean engagement levels of 8.53 out of 10 (*SD* =1.08, range = 7–10). All participants reported satisfaction with their involvement in the iPeer2Peer program and all stated that they would recommend the program to another youth living with JIA. Participants most enjoyed a) meeting someone with JIA who they could relate to, b) meeting someone older who has already experienced what they are going through (both JIA-related and non-JIA related), c) having someone to talk to, and d) getting information about JIA. See Table 2 for sample quotations from participants describing the perceived benefits of the iPeer2Peer Program.

**Table 2:** Sample quotations from participant post-iPeer2Peer program interviews

Female; 14 years old	*I liked that you are talking to someone who was older than you and who had already gone through high school, which I’m just starting now.*
Female; 13 years old	*It was good because I’ve never talked to anyone close to my age about my arthritis. Um, so, that was interesting. And then like we had a lot in common about it. And then it was good cause like she gave me some information because I didn’t really know exactly what it – what arthritis is. So she talked a little bit about it to me and then she gave me some links…and I read about it on the Internet.*
Female; 15 years old	*I liked, what I liked was that I was able to talk about school because I was heading to grade 12 next year and like by then like I wanted to make sure what I was doing, like when I was going into university and knowing what I needed to have and stuff. And so she was already in college so it was kind of good to know that someone like knows what they are doing and could help me.*
Female; 12 years old	*The thing I liked the best was that, um, I had someone I could look up to and um, I could tell myself that if I stick with the route I'm going with arthritis I could turn it be like with the same courage. I would also be able to be determined to say that there is nothing that could stop me from doing whatever I want.*
Male; 16 years old	*I think what I liked best was that I had someone who I could talk to about my arthritis who has had it before. I learned a little more about my arthritis, which I didn’t know so that was good because I actually went on a website and looked it up.*

### Secondary outcome analysis

Marginal models were computed to examine group differences on each secondary outcome including pain, self-efficacy, HRQL and self-management. There were no significant group differences on secondary outcome measures at baseline. After controlling for baseline self-management score, adolescents in the intervention group had higher self-management scores after the iPeer2Peer program compared to those in the control group (*Z* = 2.11, *p* < 0.05, d = 0.72). There were no significant differences between groups in ratings of pain, self-efficacy, or any domains of HRQL at study completion. See Table 3 for the comparison of secondary outcome measures between the two groups.

**Table 3:** Secondary outcomes for participants by treatment condition

Variable	Experimental group	Control group	Test statistics |Z|	*p*	Effect size
	Mean (SD)	Mean (SD)			(Cohen’s d)
	(*n* = 18)	(*n* = 14)			
Pain Intensity (average; range = 0–10, higher scores = higher pain intensity)			0.48	0.63	0.06
Pre-treatment	2.39 (2.06)	2.43 (2.65)			
Post-treatment	2.06 (1.83)	2.21 (1.97)			
Self-Efficacy (CASES; range = 11–55, higher scores = greater efficacy)			0.07	0.95	0.29
Pre-treatment	37.28 (12.22)	39.57 (11.10)			
Post-treatment	40.28 (12.52)	40.07 (12.83)			
PedsQL pain (range = 0–100, higher scores = lower problems)			0.63	0.53	0.04
Pre-treatment	65.63 (21.68)	61.16 (26.19)			
Post-treatment	67.36 (22.84)	62.05 (28.95)			
PedsQL activities (range = 0–100, higher scores = lower problems)			0.64	0.76	0.07
Pre-treatment	87.50 (20.81)	87.14 (22.08)			
Post-treatment	88.06 (18.88)	86.79 (22.33)			
PedsQL tx (range = 0–100, higher scores = lower problems)			0.98	0.33	0.33
Pre-treatment	72.22 (21.69)	73.98 (14.25)			
Post-treatment	70.44 (17.62)	76.28 (15.51)			
PedsQL worry (range = 0–100, higher scores = lower problems)			0.34	0.73	0.19
Pre-treatment	62.50 (29.32)	55.95 (23.89)			
Post-treatment	62.96 (29.60)	60.71 (30.74)			
PedsQL comm (range = 0–100, higher scores = lower problems)			0.33	0.74	0.23
Pre-treatment	63.89 (26.51)	62.50 (19.27)			
Post-treatment	63.89 (30.65)	67.26 (24.78)			
MEPS_total (range = 0–230, higher scores = higher perceived ability)			2.10	0.036	0.72
Pre-treatment	130.56 (36.98)	137.64 (26.58)			
Post-treatment	140.50 (30.31)	128.57 (30.93)			
MEPS_knowledge (range = 0–90, higher scores = higher perceived ability)			1.47	0.14	0.28
Pre-treatment	45.56 (19.17)	45.57 (15.55)			
Post-treatment	47.78 (15.39)	44.00 (12.34)			
MEPS_exercise (range = 0–40, higher scores = higher perceived ability)			0.56	0.53	0.51
Pre-treatment	26.50 (9.34)	31.57 (8.81)			
Post-treatment	27.56 (8.23)	28.79 (10.70)			
MEPS_pain (range = 0–60, higher scores = higher perceived ability)			2.11	0.035	0.74
Pre-treatment	37.72 (11.87)	40.29 (11.45)			
Post-treatment	41.22 (12.24)	35.50 (12.83)			
MEPS_social (range = 0–40, higher scores = higher perceived ability)			1.28	0.20	0.38
Pre-treatment	20.78 (6.37)	20.21 (4.81)			
Post-treatment	23.94 (6.49)	20.29 (6.91)			

*Note.* Analysis has been adjusted to account for period of observation and baseline scores of the same measure

## Discussion

The iPeer2Peer Program demonstrated acceptability and feasibility in a sub-sample of adolescents with JIA. In our sample of adolescents, many had few risk factors and/or inactive disease and did not show interest in being matched to a peer mentor in a structured and time intensive manner (e.g., 1–2 calls a week for 2 months). This suggests the iPeer2Peer Program may not be appropriate in its current form for all youth with JIA but instead a specific sub-sample of adolescents who are in need of individualized social support (e.g., whether due to poor social supports, active disease, challenging symptoms). The iPeer2Peer Program would benefit from increased flexibility in the number of calls and the length of the program in order to increase acceptability to larger proportions of adolescent JIA populations. Adolescents sought support for both JIA-related and non-JIA-related issues from their mentors. Secondary outcome analysis indicated significant improvements in adolescents’ perceived ability to manage JIA after participating in the iPeer2Peer Program.

The findings of this pilot study highlight the need for flexibility and individualization of the iPeer2Peer Program. Only 50 % of participants were able to complete ten calls within 8 weeks as suggested. However, call length was consistently twice as long as requested by the study parameters and adolescents reported enjoying the program. Moreover, there were no differences in call length between participants who completed all 10 calls and those who did not. Perhaps deviations from the suggested call schedule in the iPeer2Peer Program (e.g., number of calls, length of time between calls) is more reflective of the need for flexible individualized peer mentoring program as mentees have differing mentoring needs and desires. This is in keeping with the findings of our systematic review of peer support interventions for youth with chronic disease [14]. Peer support interventions in the systematic review varied significantly with respect to number of peer contacts, length of the program, and focus of the intervention (e.g., disease education, group activity, 1:1 mentoring). Based on this systematic review as well as the findings of this pilot study, we recommend future studies allow participants to choose between five and ten calls that can be completed in up to 12 weeks for 10 calls. Of note, peer mentoring differs from other psychosocial interventions in that it does not involve a standardized intervention protocol with respect to call content but instead a standardized peer mentor training. This allows peer mentors to address the needs of the individual adolescent. Peer mentor training will be adapted to include modules related to individualization of the iPeer2Peer Program (e.g., goals of participation, frequency of calls). Future studies of the iPeer2Peer Program with larger sample sizes should ensure post-participation outcome measurement in all adolescent participants in order to measure whether there is a dose effect of peer mentoring. Future work should also explore how the iPeer2Peer Program peer mentor training could be utilized in other contexts, such as training JIA camp counselors.

The call content of the mentoring calls varied across both JIA-related issues and general life issues. For example, adolescents sought support for transition to adult health care, and also for transition to post-secondary education. Adolescents shared many aspects of their life, not only JIA related concerns and issues. This finding is in contrast to adolescents living with chronic pain who completed the iPeer2Peer Program who focused mostly on pain-related topics [13]. In comparison to that group, conversations between adolescents and mentors with JIA were more evenly distributed between disease-related and non-disease-related topics. However, every call did involve conversation related to JIA. Adolescents with JIA in this sample had fairly low pain and disease activity levels which may have resulted in more conversations about life outside of their disease. Peer mentor training is focused on the practical process of and how to help adolescents come to their own decisions (e.g., helping develop SMART goals instead of providing solutions). This allowed the peer mentors to discuss issues unrelated to JIA and is an important part of the mentor role of being supportive to the whole person. Providing support to the whole adolescent can address physical, emotional, social and role challenges which in turn positively impacting HRQL across many domains.

Participants enrolled in the iPeer2Peer Program were mostly female. Although JIA clinical populations tend to be prominently female, the number of females recruited was over and above what would be expected based on clinical referrals or prevalence. This might be indicative of a gender difference in interest in the iPeer2Peer Program. The male participants who were enrolled in the program completed fewer than the 10 calls suggested. Male call content also differed from female call content as males tended to be factual and many conversations were in the form of questions and answers related to JIA disease information, medications, procedures and experiences. Male adolescents may prefer less intensive peer mentoring interventions or interventions that are geared towards having specific questions answered as opposed to personal sharing, which is typically uncharacteristic of male adolescent peer relationships [27]. Future work is needed to determine the preferences of adolescent males with respect to type of peer mentor and type of program (e.g., drop-in format, fewer sessions, group format, instant messaging). This further emphasizes that individualization is an essential component of effective peer mentorship programs like the iPeer2Peer Program.

The iPeer2Peer Program is unique in the use of online Skype video calls to connect adolescents to their peer mentors. Research in adult mentorship for vulnerable and underprivileged youth via email alone found that deep connections between youth and mentors were rare [28]. Research in youth with disabilities found that online mentoring via email was only partially successful [29]. Upon investigation, unsuccessful pairs used a formal tone in their writing while successful pairs were more informal and supportive [30]. By using video calls, the iPeer2Peer Program circumvents this issue as adolescents meet virtually face-to-face with their mentors allowing for a casual conversational tone. Providing the face-to-face interactions meant that adolescents were able to connect and develop relationships with their mentors that would otherwise be challenging when not in “real-time”. However, using online video calls is not without its challenges, as it requires that adolescents and their mentors schedule their time. The majority of missed or rescheduled calls from this study were the result of tests, assignments and exams. As tests, assignments and exams are a normal part of life for both adolescents and young adult mentors, increasing peer mentor training surrounding issues of scheduling calls and ensuring protected time for calls is warranted. In addition, although only three eligible patients were not able to participate because of insufficient Internet access, this does remind us that there are still adolescents with poor or no Internet access. These young people may be particularly vulnerable and feel even more isolated than those with JIA who have more ability to connect with the world through the Internet. This is particularly relevant in today’s youth given the ubiquitous nature of smartphones and social media use. In future, the iPeer2Peer Program will be offered via telephone to these youth to ensure they have access to a peer mentor.

Results of this pilot study must be interpreted in light of several limitations. Despite favorable findings, sample size in this study was small, secondary analyses were underpowered, and multiple testing corrections were not applied thus significant p-values should be reviewed with caution. The limited sample size also prevented any examinations of outcomes by cultural or socio-economic status as well as mentor quality to account for differences between mentors. Future full scale RCTs with adequate sample sizes could address these limitations. Moreover, recruitment was limited to one tertiary care pediatric hospital thus limiting the generalizability of the results. The sample was also mostly female and mean disease activity ratings were low suggesting some adolescents may not have had active disease. This may, in part, be reflective of Research Ethics Board (REB) protections put in place by the hospital that prevents patients from being approached by numerous studies such that not all eligible participants were approached by our research team (e.g., another ongoing study involving a self-management intervention limited our ability to approach many patients). Nevertheless, more work is needed to determine whether gender based and disease based (e.g., disease severity, newly diagnosed) differences exist and how best to adapt the program for all youth with JIA. The REB at the tertiary care hospital also stipulates that a member of the health care team must first approach potential participants before a research assistant can introduce the study. This precaution may have resulted in potential participants being labeled by the health care team as ineligible for reasons such as personality (e.g., shy, unreliable), inactive JIA, or patient transitioning to a community clinic. This may have inadvertently inflated our number of ‘ineligible’ participants. Call log data did not take into account the length of time participant-mentor pairings spoke on each specific topic. Future work would benefit from detailed analysis of call log data. Lastly, this pilot study did not incorporate a cost-benefit analysis in order to determine whether peer mentoring is cost effective. The cost-benefit analysis would need to consider not only the mentor time commitment but also the staff requirements with respects to ongoing day-to-day management of the iPeer2Peer Program (e.g., recruiting and training mentors, matching mentors to adolescents, offering support when needed with scheduling).

## Conclusions

In conclusion, the iPeer2Peer Program is a promising peer mentoring program for adolescents with JIA. Adolescents who completed the iPeer2Peer Program demonstrated significant improvements in self-management skills compared to controls. Adaptations to the iPeer2Peer Program to increase flexibility (e.g., number of calls, program length) as well as additions to the peer mentor training (e.g., determining goals, scheduling calls) are necessary to increase acceptability to larger proportions of adolescents with JIA. Qualitative work is also needed in order to determine necessary modifications to increase male adolescents’ interest in the iPeer2Peer Program. An RCT with adequate sample size is required to determine overall effectiveness and potential healthcare cost savings of the iPeer2Peer Program.

## Abbreviations

JIA: Juvenile Idiopathic Arthritis
HRQL: Health-related quality of life
RCT: Randomized controlled trial
ILAR: International League of Associations for Rheumatology
CRPC: Clinical research project coordinator
MEPS: Medical issues, exercise, pain and social support questionnaire
RPI: Recalled pain inventory
CASES: Children’s arthritis self-efficacy
